# Scientific collaborations and research trends in Parent-Child Interaction Therapy: a bibliometric analysis

**DOI:** 10.3389/fpsyg.2023.1167937

**Published:** 2023-05-12

**Authors:** Sümeyye Ulaş, İsmail Seçer, Erinn J. Victory, Cheryl B. McNeil

**Affiliations:** ^1^Laboratory Department of Psychological Counseling and Guidance, Atatürk University, Erzurum, Türkiye; ^2^Department of Psychology, West Virginia University, Morgantown, WV, United States; ^3^Department of Psychiatry, University of Florida, Gainesville, FL, United States

**Keywords:** parent–child interaction therapy, PCIT, bibliometric analysis, scientific collaborations, research trends

## Abstract

Parent–child interaction therapy (PCIT) is considered to be an effective intervention for children aged 2–7 years with conduct problems. PCIT research has been conducted for approximately 50 years; however, an analysis of general research patterns has not been published. In this context, the present study outlines a bibliometric analysis of scientific collaborations, prevalence across locations on the basis of countries and organizations, leading researchers, and trends within PCIT research. Findings demonstrate that PCIT is an area in which international scientific collaborations are intense and current, and collaborations continue to be formed around the world. Additionally, results indicate that dissemination of intercultural PCIT adaptations are continuous.

## 1. Introduction

Parent–child interaction therapy (PCIT) is a short-term, performance-oriented, low-cost, early intervention treatment approach that focuses on interactions between parents and children and aims to improve child behavior (Eyberg, 1998; McNeil and Hembree-Kigin, 2010). Developed by Dr. Sheila Eyberg, PCIT is a behavioral parent training program for caregivers and their children aged 2–7 years with disruptive behavior problems in which families participate in coaching sessions of play-based therapy techniques (McNeil and Hembree-Kigin, 2010). PCIT is uniquely both a systematic and experiential therapy approach as treatment is administered through live coaching of parent–child interactions (Lieneman et al., 2017). Although there are many parent-focused education programs in the literature, PCIT uniquely consists of two treatment phases based on Baumrind’s theory of authoritative parenting: Child-Directed Interaction (CDI) and Parent-Directed Interaction (PDI) (Baumrind, 1966; Eyberg and Funderburk, 2011; Furuzawa et al., 2020). CDI focuses on enhancing the parent–child relationship and increasing positive parenting skills through child-led play, whereas PDI focuses on addressing child problem behaviors through structured discipline techniques. Since the explanation of the PCIT process and techniques are not within the scope of this study, the books PCIT (McNeil and Hembree-Kigin, 2010), Handbook of Parent–Child Interaction Therapy Innovations and Applications for Research and Practice (Niec, 2018) may be consulted for further information.

Researchers have investigated PCIT as an effective treatment for a variety of emotional and behavioral concerns such as childhood depression (Luby et al., 2020; Donohue et al., 2021; Luby et al., 2021; Whalen et al., 2021), ADHD (Chronis-Tuscano et al., 2016; Leung et al., 2017; Hosogane et al., 2018), autism spectrum disorder (Hansen and Shillingsburg, 2016; Zlomke et al., 2017) language and speech disorders (Allen and Marshall, 2011; Falkus et al., 2016), and conduct disorders (Agazzi et al., 2020; Fleming et al., 2021).

Many studies examining PCIT as a treatment for emotional and behavioral concerns have found promising results. For instance, PCIT has been found to be an effective intervention for oppositional defiant disorder (ODD). ODD is often characterized by a child’s lack of respect for authority figures, and a child with a diagnosis of ODD may exhibit behaviors such as breaking the rules, tantrumming, arguing with adults, displaying provocative behaviors, and acting stubborn (American Psychiatric Association, 2013). Bagner et al. (2004) reported that PCIT implementation to a four-year-old child diagnosed with ODD was effective in reducing the child’s externalizing symptoms. Matos et al. (2009) also reported a significant decrease in child defiant behaviors following PCIT.

Additionally, a review of PCIT outcome studies measuring the effectiveness of PCIT in the rehabilitation of ADHD, one of the most common behavioral problems in children aged 2–7 years, reported that PCIT is a very effective approach in reducing ADHD symptoms (Wagner and McNeil, 2008). More recently, Matos et al. (2009), conducted a pilot study to evaluate the efficacy of PCIT in reducing ADHD symptoms. Findings demonstrated significant improvements in pretreatment ADHD hyperactivity and inattention scores across 20 children who participated in the PCIT intervention.

There are also studies demonstrating that PCIT remains effective for children who have experienced trauma. Research has found that PCIT and PCIT-based interventions are an effective approach in the rehabilitation of children who have been physically and emotionally abused, and thus, PCIT can prevent disruptive behaviors that may occur later in life (Chaffin et al., 2004; Kamo, 2010). A study conducted by Timmer et al. (2010) suggests that PCIT can be effective in developing positive parenting skills as well as effective coping and self-regulation skills in children who are victims of trauma. In further support of these findings, Pearl et al. (2012) reported that PCIT had a significant effect in reducing trauma-related symptoms in child trauma survivors and parent stress levels.

However, researchers have noted the importance of treatment completion in family outcomes (Lieneman et al., 2019), and PCIT has shown to have attrition rates ranging from 12 to 67% (Phillips et al., 2008; Lyon and Budd, 2010; Danko et al., 2016). PCIT attrition rates have been attributed to various factors such as lower socioeconomic status, younger age of the participating caregiver or child, lower caregiver education, lower levels of positive parenting skills, and higher caregiver stress (Lieneman et al., 2017). Given these findings, researchers are investigating solutions to reduce barriers to treatment.

In the international literature, there are many clinical case studies, single-subject design studies, and randomized controlled trials using PCIT as a treatment for disruptive behavior of children with autism spectrum disorder (Solomon et al., 2008; Hatamzadeh et al., 2010; Agazzi et al., 2013; Armstrong and Kimonis, 2013; Lesack et al., 2014; Armstrong et al., 2015; Masse et al., 2016; Allen et al., 2022; Han et al., 2022). Studies show that PCIT may successfully improve externalizing behavior problems and socio-emotional reciprocity of children with ASD who demonstrate varying levels of cognitive and social functionality (Ginn et al., 2017; Scudder et al., 2019). In addition to reducing problem behaviors, research supports that PCIT improves child self-esteem (Eisenstadt et al., 1993), encourages speech and language development, and teaches awareness of emotions (McElreath and Eisenstadt, 1994). These findings are noteworthy in that these improvements are intended outcomes of interventions for high-functioning children on the autism spectrum (Hatamzadeh et al., 2010). Recent research findings indicate that PCIT is important for parents’ emotion regulation in addition to managing children’s problem behaviors. As a result of his case study, Papadopoulos (2020) found that while PCIT reduced disruptive behaviors in a child with autism, treatment was also effective in reducing parental stress and improving parenting skills. In addition, treatment gains were maintained at 2–3-month follow-up assessments. Cambric and Agazzi (2019), reported that PCIT with a family of a 7-year-old boy diagnosed with high-functioning autism and ADHD resulted in improved positive parenting skills, parental use of effective commands, child adaptability, and disruptive behavior. In a systematic review conducted by Vetter (2018), it was concluded that PCIT is an effective intervention for autism spectrum disorder and attention deficit hyperactivity disorder.

The purpose of the present study is to identify scientific collaborations within the PCIT subject area, examine the prevalence of PCIT studies across locations on the basis of countries and organizations, identify leading researchers, and to reveal trends within the PCIT research through a bibliometric analysis. In this context, the research questions to be answered are as follows.

- What is the distribution of publications on PCIT by years?
- What is the distribution of publications on PCIT according to Web of Science categories?
- What is the distribution of publications on PCIT according to the publisher?
- What is the distribution of publications on PCIT according to Web of Science index?
- What is the distribution of publications on PCIT by country?
- What is the distribution of publications on PCIT by organization?
- What is the distribution of authors publishing on PCIT?
- What is the distribution of journals in which PCIT studies are published?
- What is the distribution of citations on PCIT studies?
- What are the scientific collaborations and research trends within PCIT research?

## 2. Materials and methods

The present study focuses on examining publications on PCIT through bibliometric analysis.

### 2.1. Data sources and data collection

Firstly, the bibliometric data of the data sources were searched. In this context, the Web of Science (WoS), the most prominent database of international scientific literature (Harzing and Alakangas, 2016) was searched using key search terms: (“parent child interaction”) or (“parent–child interaction”) or (“parent child interaction therapy”) or (“parent–child interaction therapy”) or (“pcit”). The searching process was completed on September 20, 2022. Keywords were searched by publication title, as the title is important for the accessibility of the studies (Bedekar and Desai, 2022). All publication years, languages, document types, and indexes were eligible for inclusion during the search. As a result, 680 publications were reached (search link).^1^

### 2.2. Data analysis

Data for the present study was taken directly from WoS. Full data records and cited references were downloaded from the WoS Export menu in a tab-delimited file format. Obtained data were analyzed bibliometrically. Depending on the purpose of determining the intellectual structure and trends of the international literature, researchers may use bibliometric analysis when the scope of the review is wide and the data sources are too large for manual review (Donthu et al., 2021). Distance-based or graphic-based mapping is preferred in bibliometric analyses. Distance-based mapping was used in this study due to focus on identifying relationships between PCIT-related items. Although there are various softwares for distance-based mapping, VOSviewer software, which is stated to have the best performance among software, was used (Van Eck et al., 2008; Artsın, 2020). In this context, with VOSviewer, data was visualized as a co-occurrence network for keywords, a bibliographic coupling network for authors and countries, and a citation network for cited authors.

## 3. Results

### 3.1. The annual publication rate of PCIT

**Figure 1:**
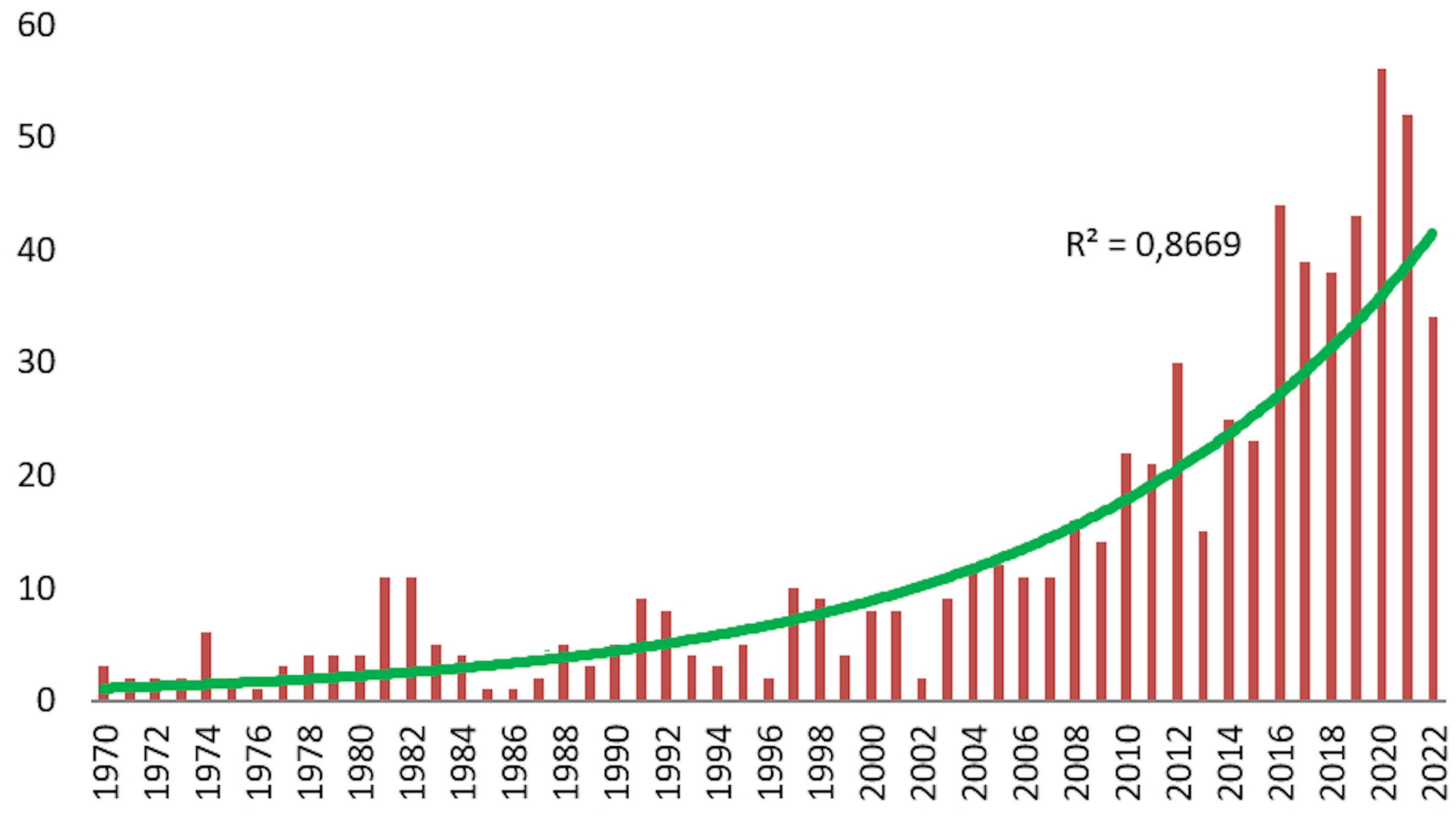
The annual publication rate of PCIT.

The number of publications in the field of PCIT provides important findings in terms of the development processes of the related field. In this context, the distribution of publications related to PCIT by year is presented in Figure 1.

According to the WoS database records, PCIT studies were first published in 1970. While in the beginning PCIT publications ranged between 1 to 5 publications per year, 1–11 publications per year were detected between 1981 and 2003, 11–30 publications per year were detected between 2004 and 2013, and 23–56 publications per year were detected between 2014 and 2022.

According to these categories of dates, each covering a range of 13 years, 8.08% of PCIT studies were published between 1970 and 1982, an additional 8.08% were published between 1983 and 1995, 18.82% were published between 1996 and 2009, and 65.02% were published between 2010 and 2022. In this context, considering the number of early publications and the number of publications in recent years, it can be determined that PCIT studies have been published with increasing intensity for 52 years.

### 3.2. Distribution of PCIT studies by WoS category

**Figure 2:**
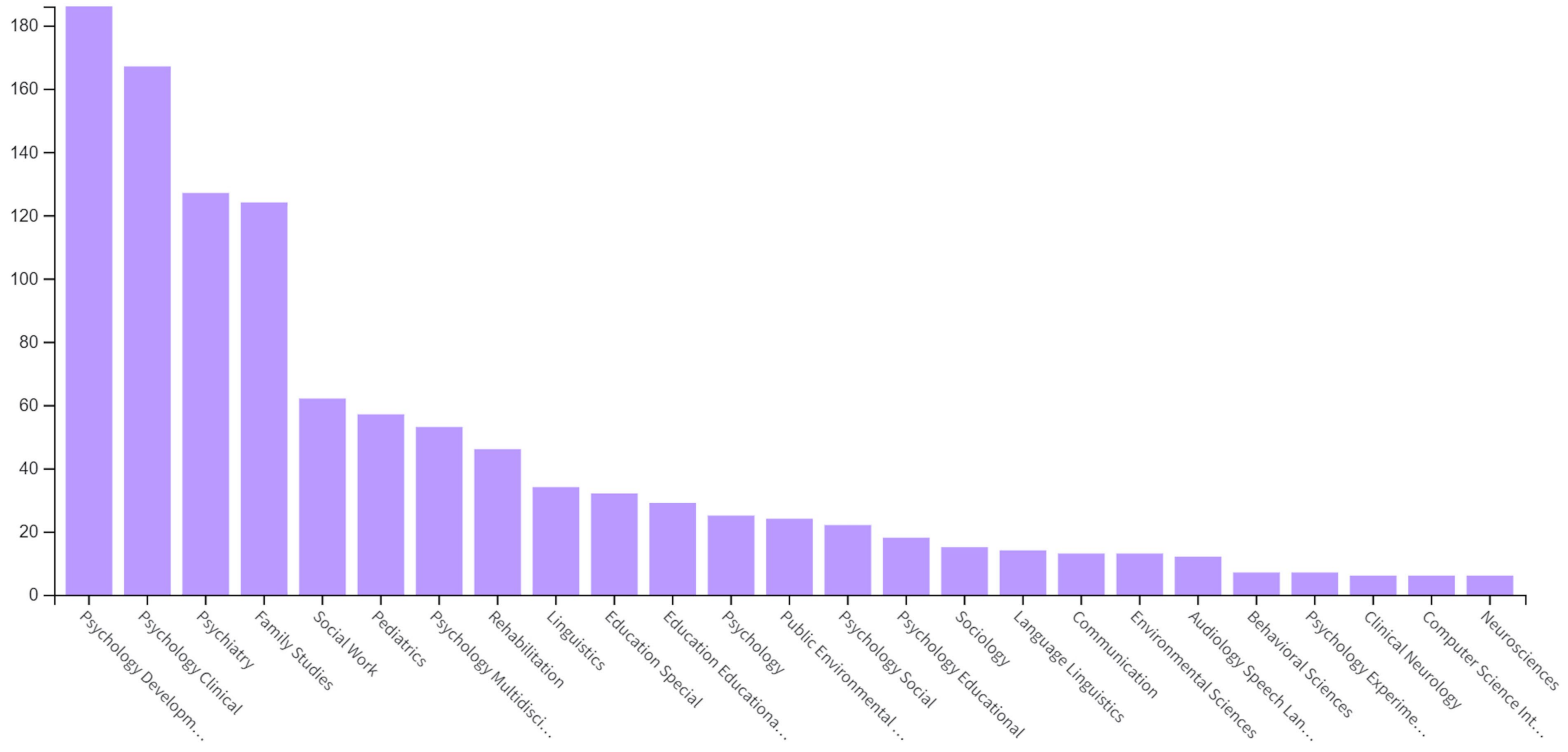
Distribution of PCIT studies by WoS category.

According to the WoS database records, PCIT studies were found to be most prominent in WoS categories of Psychology Developmental, Psychology Clinical, Psychiatry, Family Studies, Social Work, Pediatrics, Psychology Multidisciplinary, Rehabilitation, Linguistics, Education Special, Education Educational Research, Psychology, Public Environmental Occupational Health, Psychology Social, Psychology Educational, Sociology, Language Linguistics, Communication, Environmental Sciences, Audiology Speech Language Pathology, Behavioral Sciences, Psychology Experimental, Clinical Neurology, Computer Science Interdisciplinary Applications, and Neurosciences, respectively (Figure 2).

### 3.3. Distribution of PCIT studies by publisher

**Figure 3:**
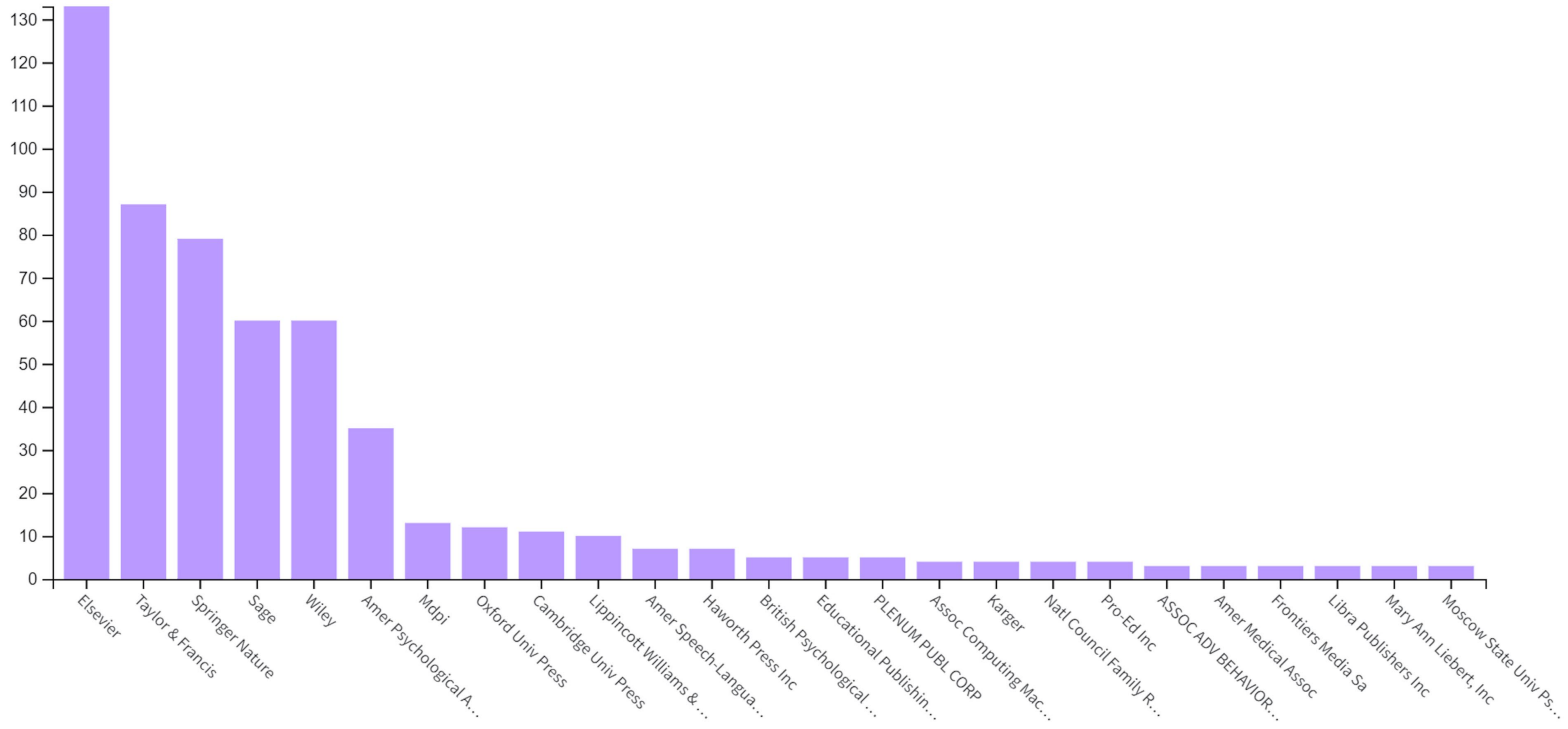
Distribution of PCIT studies by publisher.

According to WoS database records, Elsevier, Taylor & Francis, Springer Nature, Sage, Wiley, APA, MPDI, Oxford University Press, Cambridge University Press, Lippincott Williams & Wilkins, Amer Speech-Language-Hearing Assoc, Haworth Press Inc., British Psychological Soc, and Educational Publishing Foundation-American Psychological Association publish PCIT literature the most frequently (Figure 3).

### 3.4. Distribution of PCIT studies by WoS index

**Figure 4:**
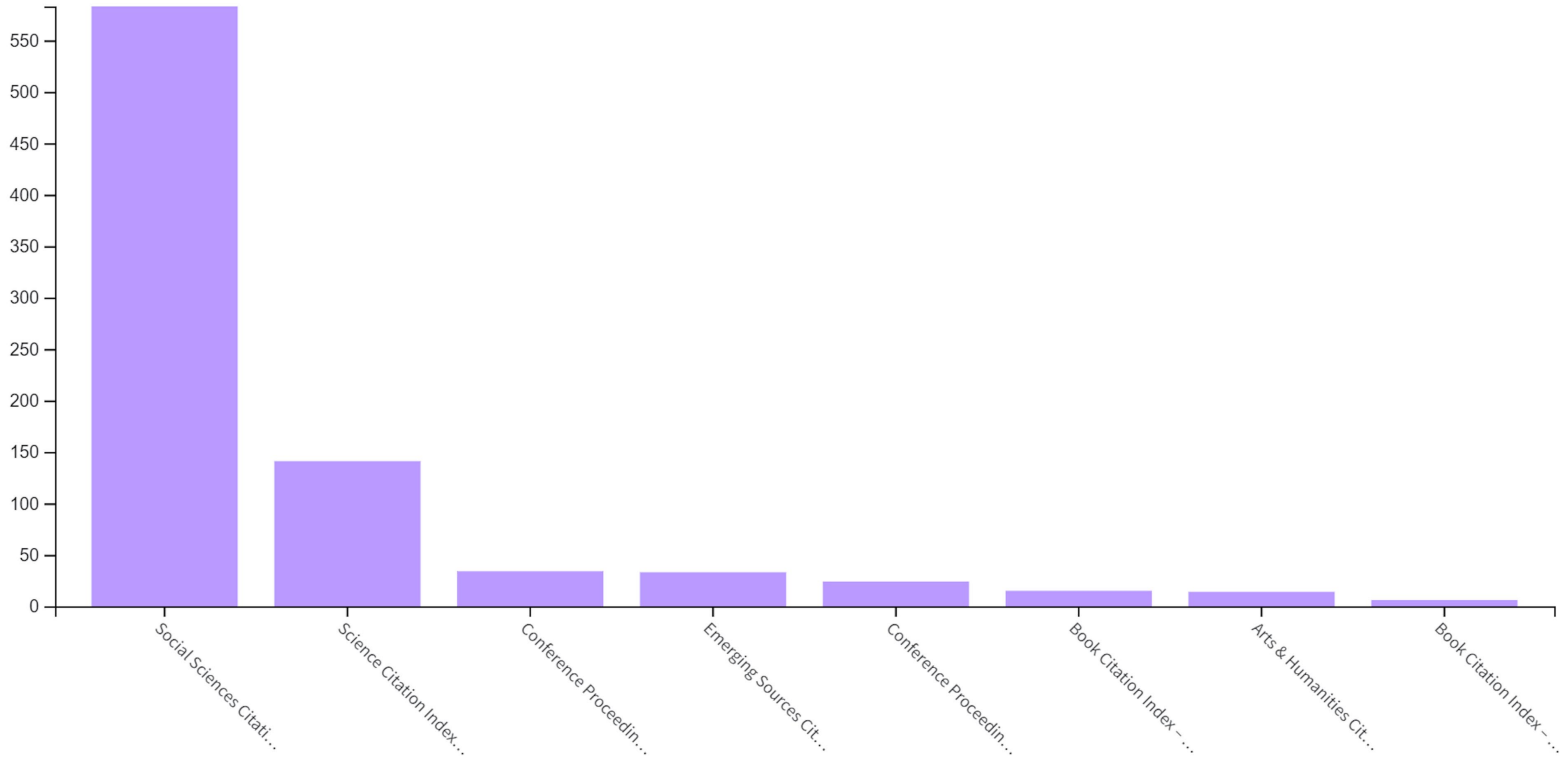
Distribution of PCIT studies by WoS index.

According to Figure 4, although most of the PCIT studies in WoS are included in the Social Sciences Citation Index (SSCI, 85.73%), there are studies in other indexes: Science Citation Index Expanded (SCI-EXPANDED, 20.73%), Conference Proceedings Citation Index-Science (CPCI-S, 5%), Emerging Sources Citation Index (ESCI, 4.85%), Conference Proceedings Citation Index – Social Science & Humanities (CPCI-SSH, 3.5%), Book Citation Index – Social Sciences & Humanities (BKCI-SSH, 2%), Arts & Humanities Citation Index (A&HCI, 2%), and Book Citation Index – Science (BKCI-S, 0.8%).

### 3.5. Country, organization, and author collaborations of PCIT studies

In the analysis carried out to determine international collaborations regarding PCIT studies, co-authorship was used as the type of analysis, countries as the unit of analysis, the minimum number of documents by country was determined as 2, and the minimum number of citations by country was determined as 2. As a result of the analysis examining over 52 countries, the countries from where PCIT studies were published were found as 20 items and 9 clusters. During the visualization process, the total link strength (tls) was used as the weight scale of each item.

**Figure 5:**
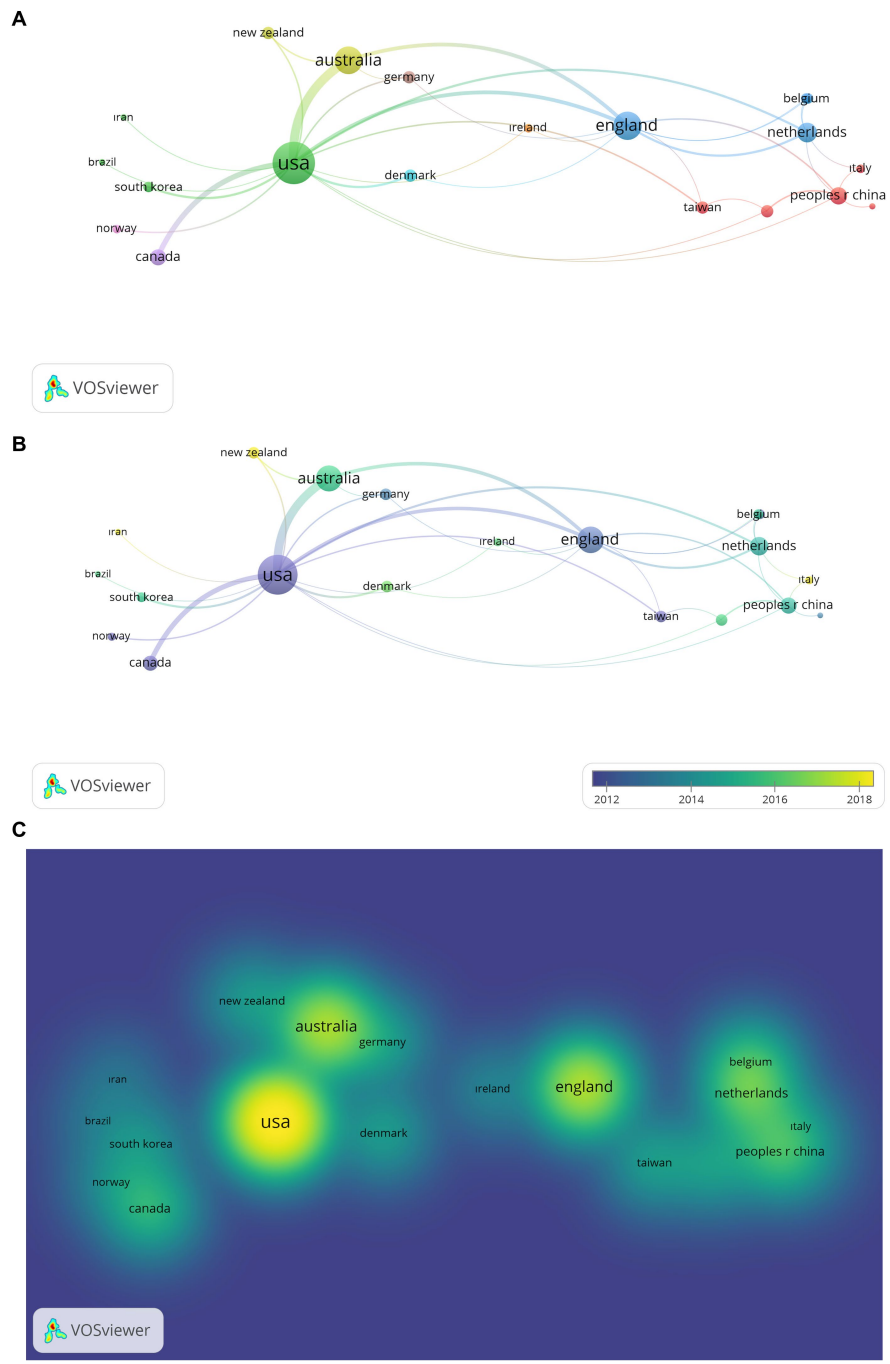
**(A)** Network Visualization of Country Collaborations of PCIT Studies. The larger the value taken according to the analysis unit, the larger the circles. The colors in the visualization do not have a specific meaning. **(B)** Overlay Visualization of Country Collaborations of PCIT Studies (Change by Years). The larger the value taken according to the analysis unit, the larger the circles. The circle colors ranging from purple to yellow indicate a chronological date. **(C)** Density Visualization of Country Collaborations of PCIT Studies. The larger the value taken according to the analysis unit, the larger the circles. The colors in the visualization do not have a specific meaning.

The first cluster, shown in Figure 5A, consists of China (tls: 8), Taiwan (tls:4), and Singapore (tls:4); the second cluster United States (tls:46), South Korea (tls:3), Brazil (tls: 1), and Iran (tls: 1); the third cluster England (tls:21), the Netherlands (tls: 10), Sweden (tls:3), and Belgium (tls:3); the fourth cluster Australia (tls: 20), and New Zealand (tls:4); the fifth cluster Canada (tls:7); the sixth cluster Denmark (tls: 4); the seventh cluster Ireland (tls:2); the eighth cluster Germany (tls:4); the ninth cluster Norway (tls:2).

According to the results of the analysis to determine the central country, it can be said that the United States leads all countries in published PCIT studies (Figure 5C), and there is intense scientific cooperation between countries. This finiding is consistent with the history of the intervention, as PCIT was developed in the United States and later expanded to countries outside of the United States (Bjørseth et al., 2010; Funderburk and Eyberg, 2011). In addition to the United States, it is seen that England and Australia comes to the fore. According to Figure 5B, it can be interpreted that Japan, Denmark, New Zealand, and Australia have established scientific collaborations in recent years.

**Figure 6:**
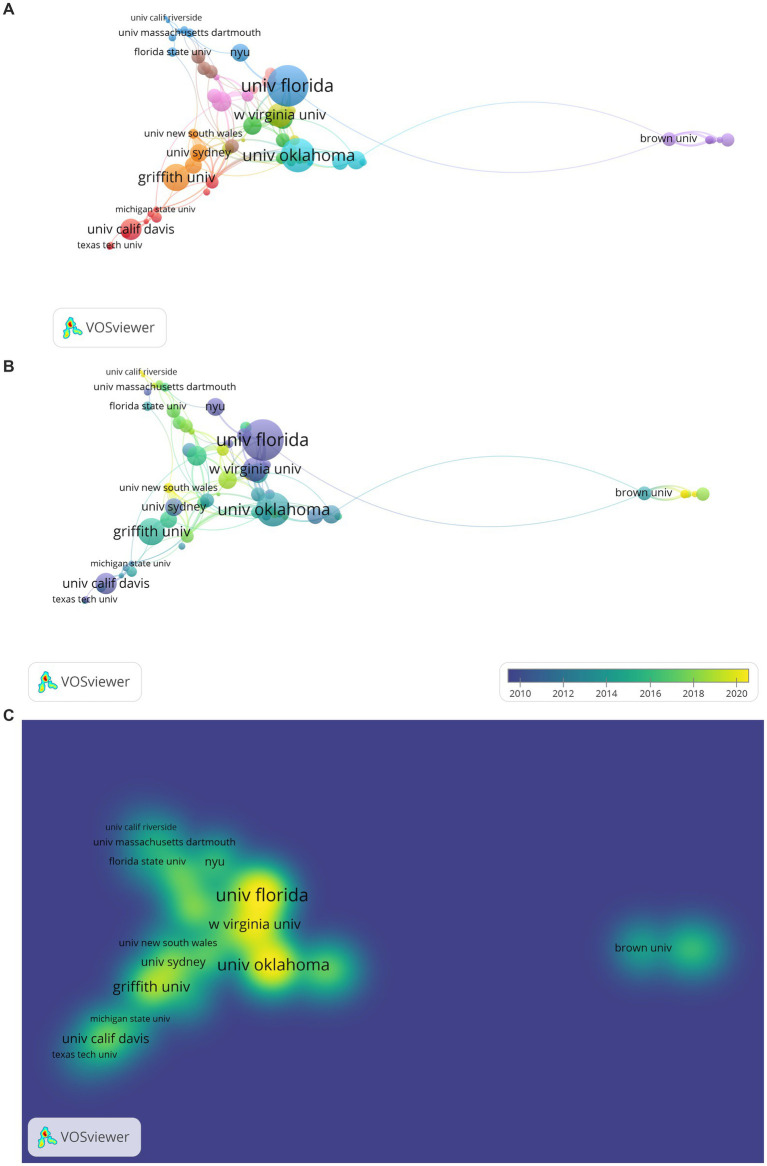
**(A)** Network Visualization of Organizational Collaborations of PCIT Studies. The larger the value taken according to the analysis unit, the larger the circles. The colors in the visualization do not have a specific meaning. **(B)** Overlay Visualization of Organizational Collaborations of PCIT Studies. The larger the value taken according to the analysis unit, the larger the circles. The circle colors ranging from purple to yellow indicate a chronological date. **(C)** Density Visualization of Organizational Collaborations of PCIT Studies. The larger the value taken according to the analysis unit, the larger the circles. The colors in the visualization do not have a specific meaning.

The result of the analysis conducted to determine the distribution and collaborations among organizations from where PCIT studies are published is presented in Figure 6A. Co-authorship was used as the type of analysis, organizations as the unit of analysis, the minimum number of documents of organizations was determined as 3, and the minimum number of citations of organizations was determined as 10. Due to these limitations, the number of organizations decreased from 680 to 89. However, in the examinations made, it was determined that there are different usages in the entrance of the same organization to WoS (e.g., West Virginia University may appear as West Virginia, W Virginia, or WVU). For this, the total link strength of the relevant organizations has been collected and presented. As a result, 65 items and 10 clusters were formed. During the visualization process, the number of citations were used for the weight scale of each item.

When Figure 6A is examined, the first cluster for organizations consists of Univ Calif Davis (citations: 451), Central Michigan Univ (citations: 172), Auburn Univ (citations: 108), UC Davis Childrens Hosp (citations: 90), Texas Tech Univ (citations: 61), Northwestern Univ (citations: 54), Univ Amsterdam (citations: 52), Michigan State Univ (citations: 40), Harvard Univ (citations: 30), and Towson Univ (citations: 28). The second cluster consists of Boston Univ (citations: 405), Florida Int. Univ (citations: 346), Colombia Univ (citations: 201), Univ Washington (citations: 382), Georgia State Univ (citations: 134), Harvard Medicine School (citations: 94), Rutgers State Univ (citations: 80), Univ Oregon (citations: 75), and Penn State (citations: 14). The third cluster consists of Univ Florida (citations: 1722), New York Univ (citations: 333), Univ Massachusetts Dartmouth (citations: 79), Univ Denver (citations: 60), Univ Delaware (citations: 59), Boston Child Study Center (citations: 35), Univ Arkansas (citations: 20), and Univ Calif Riverside (citations: 19). The fourth cluster consists of West Virginia Univ (citations: 846), Univ State Florida (citations: 246), Univ Pittsburgh (citations:135), Calif State Univ Sacramente (citations: 103), Early Childhood Mental Health Services (citations:84), Arizona State Univ (citations:64), Johns Hopkins Univ (citations: 17), and Iowa State Univ (citations: 11). The fifth cluster consists of Brown Univ (citations: 192), Univ Calif Santa Cruz (citations: 78), New York Hall SCI (citations: 78), Emory Univ (citations: 48), and Univ Texas Austin (citations: 46). The sixth cluster consists of Univ Oklahoma (citations: 1183), Univ Michigan (citations: 345), Center for Disease Control and Prevention (citations: 252), Cincinnati Children Hospital Medical Center (citations: 60), and Univ S Alabama (citations: 40). The seventh cluster consists of Griffith Univ (citations: 759), Univ Sydney (citations: 320), Bond Univ (citations: 286), Univ New South Wales (citations: 102), and Karitane (citations: 76). The eighth cluster consists of Univ Calif San Diego (citations: 203), Univ Calif Los Angeles (citations: 198), San Diego State Univ (citations:189), San Diego Univ (citations: 187), and Virginia Commonwealth Univ (citations: 78). The ninth cluster consists of Univ Miami (citations: 391), Stanford Univ (citations: 219), Duke Univ (citations:143), and Univ Calif Santa Barbara (citations: 47). The tenth cluster consists of Univ Illinois (citations: 208), Univ Wisconcin (citations: 133), Univ Utah (citations: 72), and JBS International Inc., (citations: 12).

According to Figure 6C, Univ Florida, Univ Oklahoma, West Virginia University, Griffith Univ, Univ Calif Davis, New York Univ, Univ Sydney, Florida State Univ, Univ New South Wales, Univ Massachusetts Dartmouth, Texas Tech Univ, Michigan State Univ, and Univ Calif Riverside are the most prominent centers for PCIT research. Figure 6B illustrates that PCIT research from Univ New South Wales and Univ Calif Riverside has been cited the most recently.

**Figure 7:**
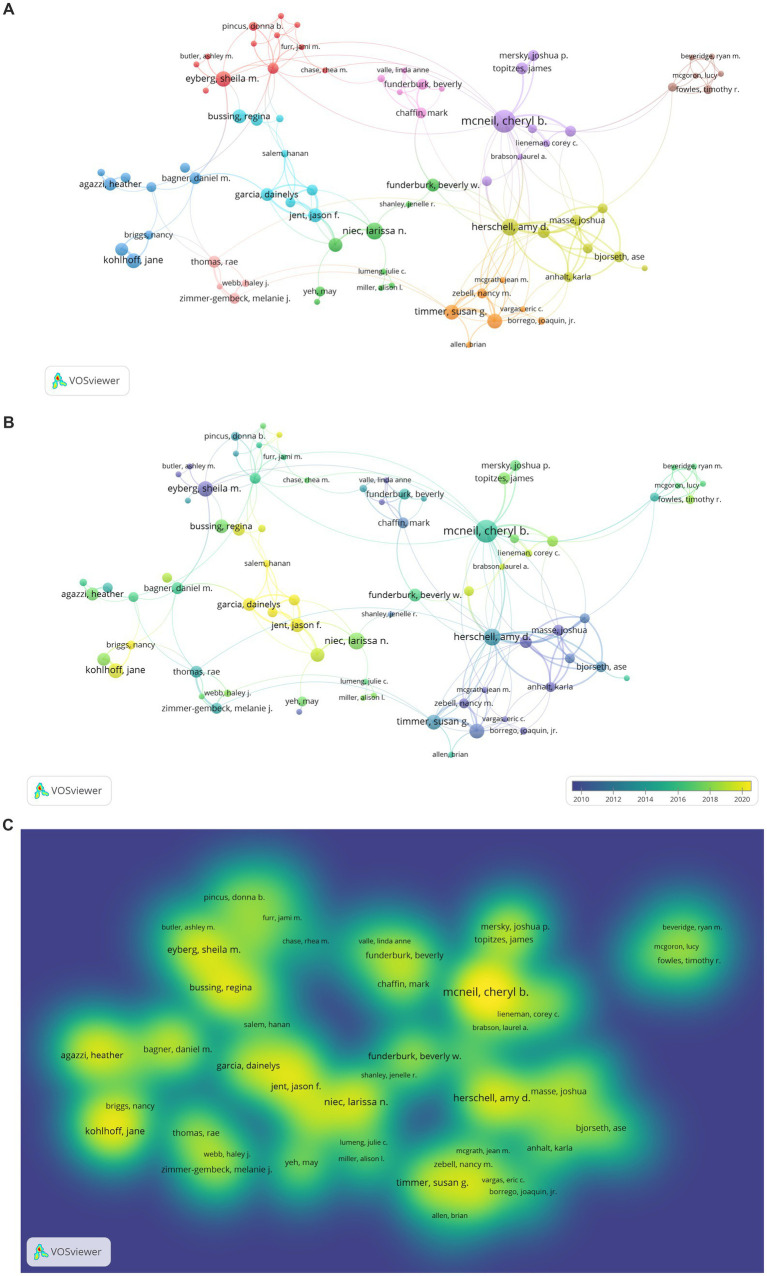
**(A)** Network Visualization of Researchers’ Collaboration in PCIT Studies. The larger the value taken according to the analysis unit, the larger the circles. The colors in the visualization do not have a specific meaning. **(B)** Overlay Visualization of Researchers’ Collaboration in PCIT Studies. The larger the value taken according to the analysis unit, the larger the circles. The circle colors ranging from purple to yellow indicate a chronological date. **(C)** Density Visualization of Researchers’ Collaboration in PCIT Studies. The larger the value taken according to the analysis unit, the larger the circles. The colors in the visualization do not have a specific meaning.

The result of the analysis examining researchers who are in scientific collaboration and relatively central positions among PCIT studies is presented in Figure 7A. The type of analysis was co-authorship, the unit of analysis was authors, the minimum number of documents by authors was 2, and the minimum number of citations by authors was 25. While there were 1895 PCIT researchers in total, the analysis was carried out with 77 researchers who were determined to meet these conditions. Due to WoS registration differences, the same investigator was visualized more than once (e.g., Eyberg, S., Eyberg, Sheila M., Eyberg, SM or McNeil Cheryl, McNeil Cheryl B, McNeil Cheryl Bodiford, McNeil CB or Funderburk Beverly, Funderburk Beverly W.). If the same researcher appeared in different clusters, the researcher was placed in the cluster with their highest values. As a result, a map with 77 items and 10 clusters was obtained.

According to Figure 7A, scientific collaborations between PCIT researchers in the first cluster includes Sheila M. Eyberg (documents: 25, tls: 23), Jonathan S. Comer (documents: 6, tls: 17), Rhea Chase (documents: 4, tls: 9), Melanie A. Fernandez (documents: 2, tls: 3), Donna B. Pincus (documents: 4, tls: 7), Anthony C. Puliafico (documents: 2, tls: 5), Aubrey I. Carpenter (documents: 2, tls: 7), Jami M. Furr (documents: 2, tls: 8), Ashley M. Butler (documents: 2, tls: 3), Meredith R. Elkins (documents: 2 tls: 5), and Kelly A. O’Brien (documents:2, tls:1). Those in the second cluster are Beverly W. Funderburk (documents: 14, tls: 18), Larissa N. Niec (documents: 13, tls: 9), Miya L. Barnett (documents: 9, tls: 12), Julie C. Lumeng (documents: 2, tls: 3), Kristen McCabe (documents: 2, tls: 2), Alison I. Miller (documents: 2, tls: 3), Katherine I. Rosenblum (documents: 2, tls: 3), Jenelle R. Shanley (documents: 2, tls: 2), and May Yeh (documents: 5, tls: 3). Those in the third cluster are Daniel M. Bagner (documents: 7, tls:8), Jane Kohlhoff (documents: 10, tls: 10), Susan Morgan (documents: 8, tls: 10), Nancy Briggs (documents: 4, tls: 9), Heather Agazzi (documents: 8, tls: 6), Kathleen Armstrong (documents: 5, tls: 5), Eva R. Kimonis (documents: 5, tls: 7), Sim Yin Tan (documents: 3, tls: 4), and Paulo A. Graziano (documents: 5, tls: 1). Those in the fourth cluster are Amy D. Herschell (documents: 13, tls: 44), Joshua Masse (documents: 10, tls: 41), Stephanie Wagner (documents: 5, tls: 27), Lisa M. Ware (documents: 7, tls: 31), Karla Anhalt (documents: 5, tls: 27), Ase Bjorseth (documents: 6, tls: 26), Yi-Chuen Chen (documents: 5, tls: 24), and Lars Wichstrom (documents: 2, tls: 2). Those in the fifth cluster are Cheryl B. McNeil (documents: 23, tls: 37), Corey C. Lieneman (documents: 3, tls: 7), Joshua P. Mersky (documents: 6, tls: 9), Lauren B. Quetsch (documents: 6, tls: 11), James Topitzes (documents: 7, tls: 10), Nancy M. Wallace (documents: 4, tls: 7), Kristen F. Schaffner (documents: 5, tls: 8), and Lauren A. Brabson. Those in the sixth cluster are Melanie M. Nelson (documents: 7, tls: 9), Dainelys Garcia (documents: 8, tls: 18), Regina Bussing (documents: 9, tls: 7), Jason F. Jent (documents: 9, tls: 24), Andrew W. Rothenberg (documents: 5, tls: 16), Hanan Salem (documents: 3, tls: 11), and Allison Weinstein (documents: 6, tls: 18). Those in the seventh cluster are Brian Allen (documents: 2, tls: 4), Joaquin Borrego (documents: 3, tls: 3), Jean M. McGrath (documents: 2, tls: 10), Susan G. Timmer (documents: 10, tls: 25), Anthony J. Urquiza (documents: 10, tls: 26), Eric C. Vangas (documents: 2, tls: 8), and Nancy M. Zebell (documents: 5, tls: 17). Those in the eighth cluster are Ryan M. Beveridge (documents: 2, tls: 10), Timothy R. Fowles (documents: 4, tls: 10), Lucy McGoron (documents: 2, tls: 10), Brendth P. Parrish (documents: 2, tls: 10), and Marissa A. Smith (documents: 2, tls: 10). Those in the ninth cluster are David Bard (documents: 2, tls: 8), Mark Chaffin (documents: 6, tls: 14), Robin H. Gurwitch (documents: 6, tls: 12), Jane F. Silovsky (documents: 2, tls: 3), and Linda Anne Valle (documents: 2, tls: 8). Those in the tenth cluster are Elbina Avdogic (documents: 2, tls: 6), Rae Thomas (documents: 7, tls: 13), Haley J. Webb (documents: 3, tls: 8), and Melanie J. Zimmer-Gembeck (documents: 6, tls: 11).

When Figures 7B,C are examined, the most prominent researchers of PCIT are Sheila Eyberg, Regina Bussing, Daniel M. Bagner, Agazzi Heather, Jane Kohlhoff, Larissa N. Niec, Susan G. Timmer, Amy D. Herschell, Joshua Masse, Beverly Funderburk, Cheryl B. McNeil, and Corey C. Lieneman.

**Figure 8:**
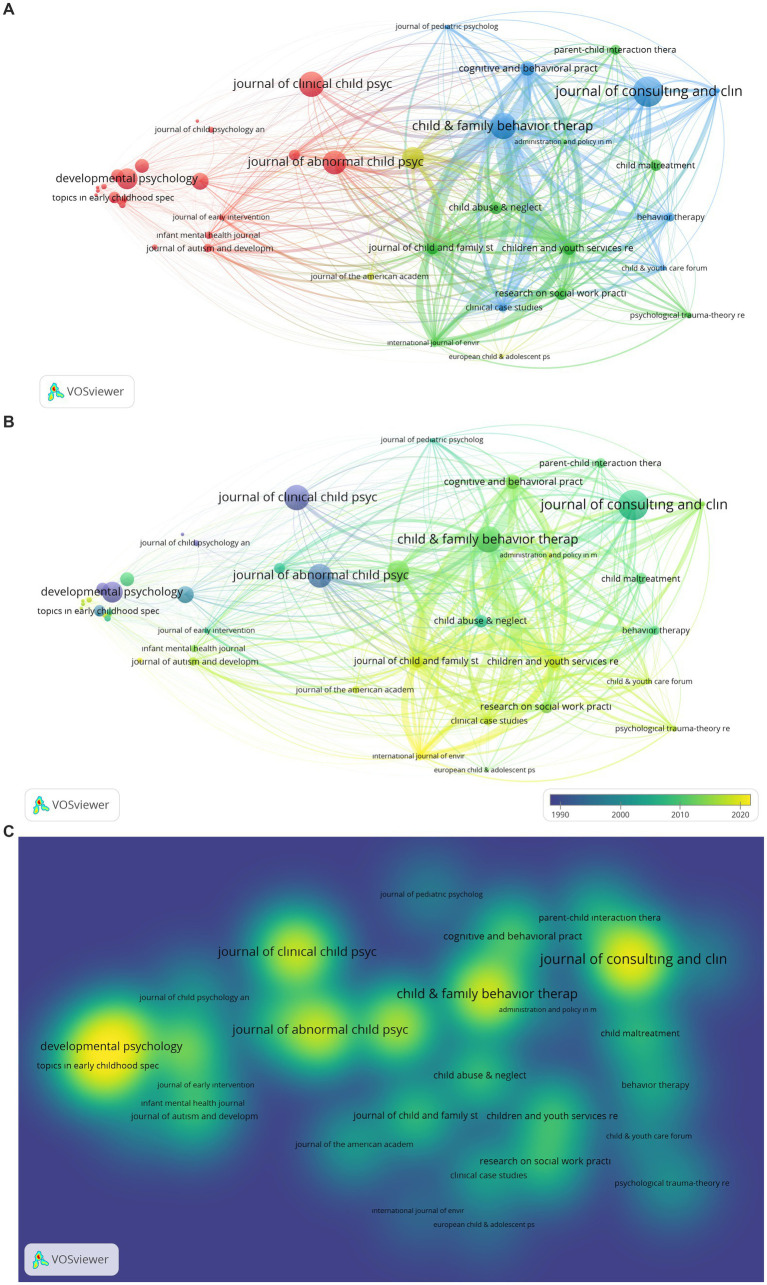
**(A)** Network Visualization of Journals Publishing PCIT Studies. The larger the value taken according to the analysis unit, the larger the circles. The colors in the visualization do not have a specific meaning. **(B)** Overlay Visualization of Journals Publishing PCIT Studies. The larger the value taken according to the analysis unit, the larger the circles. The circle colors ranging from purple to yellow indicate a chronological date. **(C)** Density Visualization of Journals Publishing PCIT Studies. The larger the value taken according to the analysis unit, the larger the circles. The colors in the visualization do not have a specific meaning.

The mapping of the analysis of journals that have published PCIT research is given in Figures 8A–C. Bibliographic coupling was used as the type of analysis, sources as the unit of analysis, the minimum number of documents of sources were determined as 3, and the minimum number of citations of sources were determined as 10. As a result of the analysis made on 330 total sources, it was found that 43 items and 4 clusters came to the fore. During the visualization, citations were used as the weight scale of the items.

Analysis results examining the journals that have published PCIT research are shown in Figures 8A–C. Journals in the first cluster are Adolescence (tls: 6, citations: 10), Child Development (tls: 666, citations: 447), Child Language Teaching & Therapy (tls: 72, citations: 49), Developmental Psychology (tls: 164, citations: 619), Early Child Development and Care (tls: 39, citations: 42), Family Process (tls: 1122, citations: 225), Infant Behavior and Development (tls: 88, citations: 76), Infant Mental Health Journal (tls: 466, citations: 123), Infant and Young Children (tls: 20, citations: 33), International Journal of Language and Communication Disorder (tls: 111, citations: 34), Journal of Abnormal Child Psychology (tls: 1929, citations: 781), Journal of Applied Developmental Psychology (tls: 64, citations: 108), Journal of Autism and Developmental Disorders (tls: 1157, citations: 148), Journal of Child Psychology and Psychiatry and Applied Disciplines (tls: 113, citations: 89), Journal of Clinical Child Psychology (tls: 781, citations: 846), Journal of Early Intervention (tls: 1049, citations: 68), Journal of Family Psychology (tls: 140, citations: 336), Journal of Marriage and Family (tls: 22, citations: 208), Journal of Pediatrics (tls: 36, citations: 78), Journal of Speech Language and Hearing Research (tls: 80, citations: 78), Research of Development Disabilities (tls: 151, citations: 83), Social Development (tls: 53, citations: 99), and Topics in Early Childhood Special Education (tls: 53, citations: 245). Those in the second cluster are Administration and Policy in Mental Health and Mental Health Services Research (tls: 985, citations: 36), Child Abuse & Neglect (tls: 2656, citations: 283), Child Maltreatment (tls: 1311, citations: 245), Children and Youth Services Review (tls: 10483, citations: 288), International Journal of Environmental Research and Public Health (tls: 4568, citations: 21), Journal of Child and Family Studies (tls: 10144, citations: 265), Psychological Trauma-Theory Research Practice and Policy (tls: 1297, citations: 94), and Research on Social Work (tls: 3302, citations: 265). Those in the third cluster are Behavior Therapy (tls: 2588, citations: 167), Child and Family Behavior Therapy (tls: 13039, citations: 909), Child and Youth Care Forum (tls: 1686, citations: 63), Clinical Case Study (tls: 5226, citations: 150), Cognitive and Behavioral Practice (tls: 5560, citations: 344), Journal of Consulting and Clinical Psychology (tls: 3189, citations: 1142), Journal of Pediatrics Psychology (tls: 1247, citations: 50), and Journal of Psychopathology and Behavioral Assessment (tls: 2363, citations: 59). Those in the fourth cluster are European Child and Adolescent Psychiatry (tls: 450, citations: 20), Journal of Clinical Child and Adolescent Psychology (tls: 3797, citations: 682), and Journal of American Academy of Child and Adolescent Psychiatry (tls: 295, citations: 130).

When Figures 8B,C are examined, it is seen that Developmental Psychology, Topics in Early Childhood Special Education, Journal of Abnormal Child Psychology, Child & Family Behavior Therapy, and Journal of Consulting and Clinical Psychology come to the fore among the journals in which PCIT research has been published.

**Figure 9:**
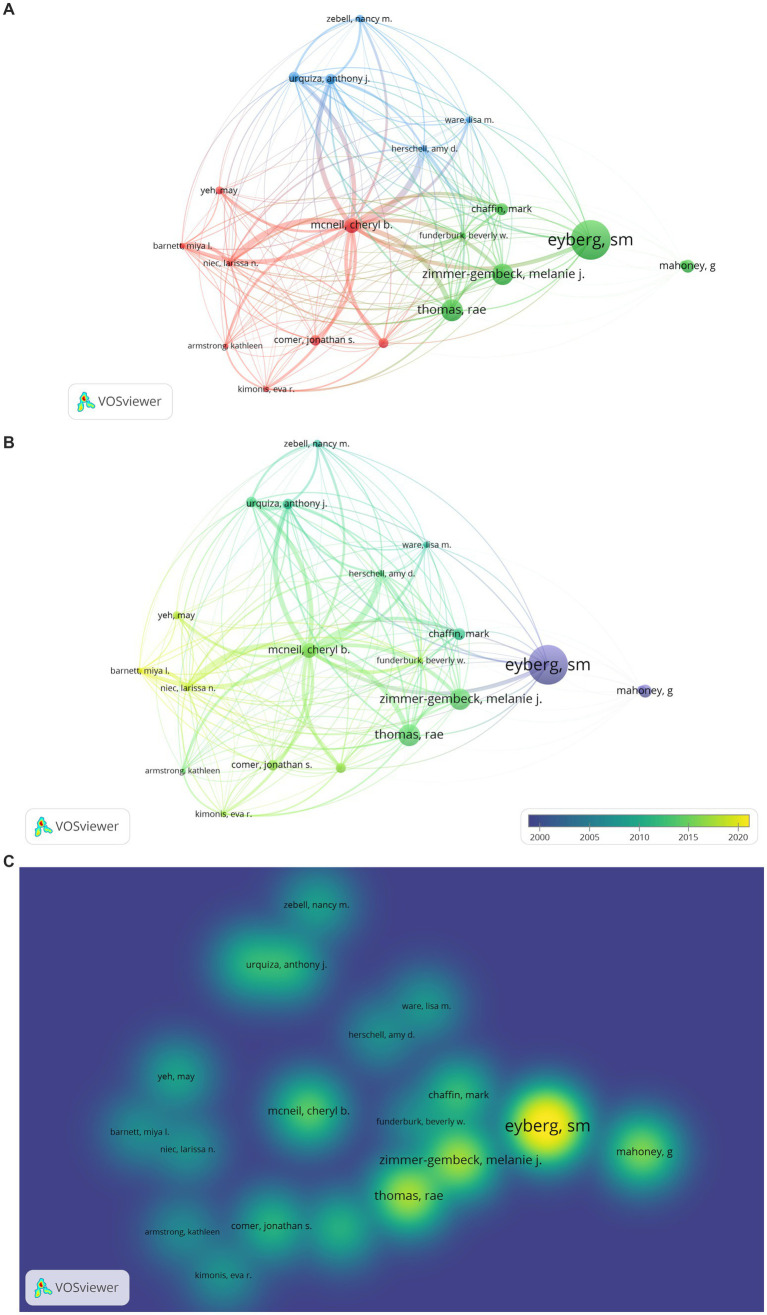
**(A)** Citation Network Visualization of PCIT Researchers. The larger the value taken according to the analysis unit, the larger the circles. The colors in the visualization do not have a specific meaning. **(B)** Citation Overlay Visualization of PCIT Researchers. The larger the value taken according to the analysis unit, the larger the circles. The circle colors ranging from purple to yellow indicate a chronological date. **(C)** Citation Density Visualization of PCIT Researchers. The larger the value taken according to the analysis unit, the larger the circles. The colors in the visualization do not have a specific meaning.

In addition, the citation network among published PCIT researchers was analyzed with the researcher’s unit and presented in Figures 9A–C. Citation was used as the type of analysis, author as a unit of analysis, the minimum number of documents of the author was determined as 5, and the minimum number of citations of the author was determined as 100. While there were 1895 PCIT researchers in total, an analysis was carried out with 20 researchers who were determined to meet these conditions. During the visualization, citations were used as the weight scale of the items.

The results of the analysis to determine the citation network for PCIT researchers are shown in Figures 9A–C. Authors appearing in the first cluster are Kathleen Armstrong (citations: 100), Daniel M. Bagner (citations: 265), Miya L. Barnett (citations: 107), Jonathan S. Comer (citations: 279), Eva R. Kimonis (citations: 128), Cheryl B. McNeil (citations: 459), Larissa N. Niec (citations: 121), and May Yeh (citations: 187). Those in the second cluster are Sheila M. Eyberg (citations: 1832), Mark Chaffin (citations: 332), Beverly W. Funderburk (citations: 114), Rae Thomas (citations: 790), Melanie J. Zimmer-Gembeck (citations: 757), H. Lyton (citations: 211), and G. Mahoney (citations: 379). Those in the third cluster are Amy D: Herschell (citations: 116), Susan G. Timmer (citations: 273), Anthony J. Urquiza (citations: 277), Lisa M. Ware (citations: 141), and Nancy M. Zebell (citations: 173).

When Figures 9B,C are examined, it can be determined that the researchers with the most citations are Sheila M. Eyberg, Melanie J. Zimmer-Gembeck, Rae Thomas, Cheryl B. McNeil, and Anthony J. Urquiza.

In a co-occurrence analysis of publications within the scope of the study, the author keywords were determined as the analysis unit. In this context, it is aimed to map the whole conceptualization by reducing the minimum number of common keywords to 2 in the study. Based on the 2-word limit, 219 words were mapped from the data set containing 991 keywords. However, as in other mappings, there are repetitive keywords (e.g., disruptive problem-disruptive problems). These are excluded in the visualizations. The visualization is based on occurrences as the weighting criterion.

**Figure 10:**
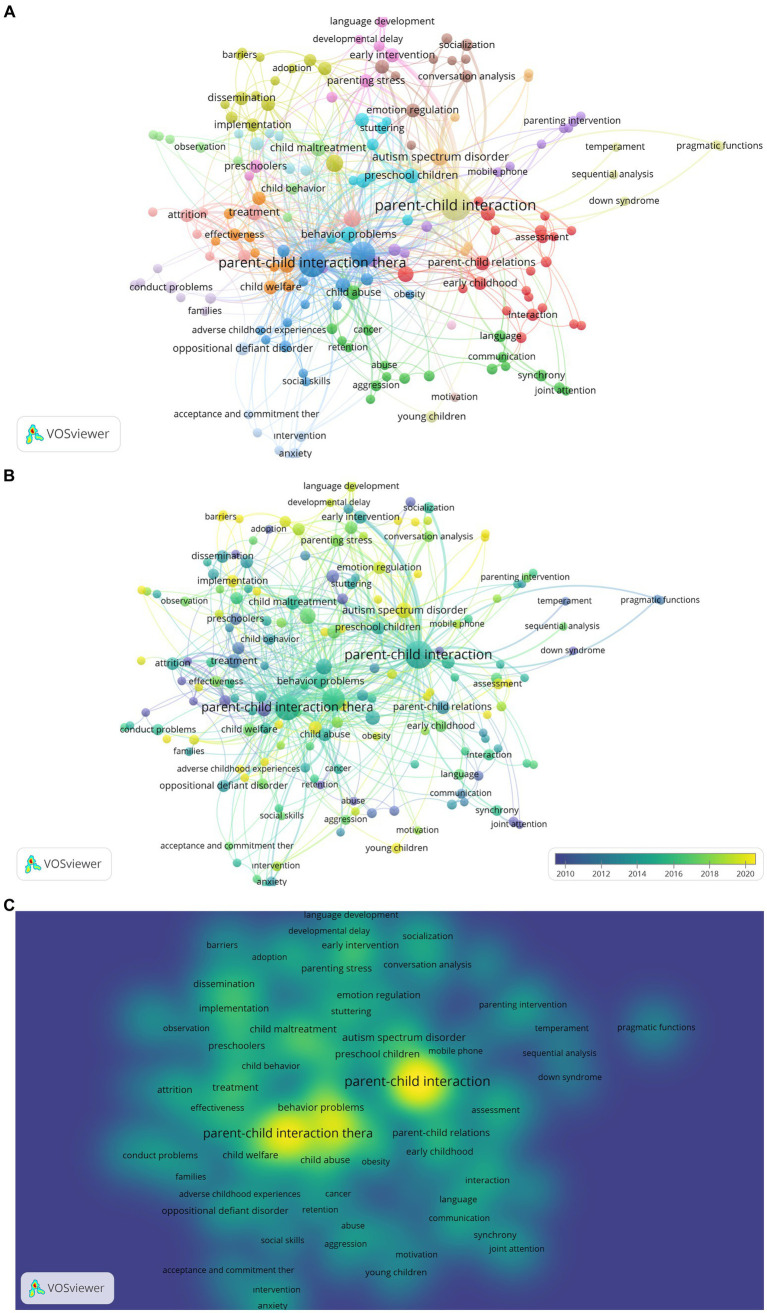
**(A)** Co-occurrence Network Visualization of Keywords of PCIT Research. The larger the value taken according to the analysis unit, the larger the circles. The colors in the visualization do not have a specific meaning. **(B)** Co-occurrence Overlay Visualization of Keywords of PCIT Research. The larger the value taken according to the analysis unit, the larger the circles. The circle colors ranging from purple to yellow indicate a chronological date. **(C)** Co-occurrence Density Visualization of Keywords of PCIT Research. The larger the value taken according to the analysis unit, the larger the circles. The colors in the visualization do not have a specific meaning.

According to Figure 10A, 172 items and 18 clusters were formed as a result of the co-occurrence analysis. The first cluster includes analogue behavior observation (tls: 3, occurrence: 2), child interaction (tls: 4, occurrence: 2), ADHD (tls: 6, occurrence: 8), deaf (tls: 6, occurrence: 4), depression (tls: 2, occurrence: 2), early childhood (tls: 11, occurrence: 6), early years (tls: 6, occurrence: 3), home learning environment (tls: 3, occurrence: 2), interaction (tls: 7, occurrence: 5), intervention (tls: 20, occurrence: 11), language delay (tls: 2, occurrence: 2), Latino families (tls: 2, occurrence: 2), mother (tls: 3, occurrence: 2), music therapy (tls: 3, occurrence: 2), parent–child relations (tls: 15, occurrence: 9), parental responsiveness (tls: 2, occurrence: 2), play (tls: 6, occurrence: 3), reading (tls: 3, occurrence: 2), school adjustment (tls: 3, occurrence: 2), self-esteem (tls: 3, occurrence: 2), and speech and language therapy (tls: 6, occurrence: 2).

Items in the second cluster are abuse (tls: 5, occurrence: 2), addiction (tls: 4, occurrence: 2), aggression (tls: 5, occurrence: 3), cancer (tls: 8, occurrence: 3), child abuse (tls: 20, occurrence: 8), child neglect (tls: 8, occurrence: 3), communication (tls: 6, occurrence: 3), development (tls: 4, occurrence: 3), domestic violence (tls: 3, occurrence: 2), family therapy (tls: 4, occurrence: 2), joint attention (tls: 2, occurrence: 2), language (tls: 6, occurrence: 4), language acquisition (tls: 4, occurrence: 3), neglect (tls: 5, occurrence: 3), parent (tls: 5, occurrence: 3), relationship (tls: 4, occurrence: 2), retention (tls: 7, occurrence: 2), and synchrony (tls: 6, occurrence: 4). Items in the third cluster are adverse child experiences (tls: 3, occurrence: 2), case report (tls: 2, occurrence: 2), case study (tls: 10, occurrence: 4), childhood obesity (tls: 8, occurrence: 2), low-income (tls: 6, occurrence: 2), obesity (tls: 4, occurrence: 2), obesity prevention (tls: 5, occurrence: 2), parenting training program (tls: 6, occurrence: 2), parent–child interaction therapy (tls: 128, occurrence: 68), parent–child relationship (tls: 12, occurrence: 5), parenting (tls: 92, occurrence: 47), self-regulation (tls: 14, occurrence: 5), social skills (tls: 2, occurrence: 2), telehealth (tls: 14, occurrence: 8), and traumatic brain injury (tls: 5, occurrence: 2). Items in the fourth cluster are adoption (tls: 2, occurrence: 2), attachment (tls: 12, occurrence: 6), barriers (tls: 4, occurrence: 2), behavioral parenting training (tls: 24, occurrence: 14), childhood conduct problems (tls: 5, occurrence: 2), consultation (tls: 4, occurrence: 2), dissemination (tls: 9, occurrence: 7), evidence based treatment (tls: 13, occurrence: 7), facilitators (tls: 4, occurrence: 2), implementation (tls: 14, occurrence: 6), parent management training (tls: 4, occurrence: 2), therapist training (tls: 4, occurrence: 2), time-out (tls: 6, occurrence: 2), and training (tls: 5, occurrence: 4). Items in the fifth cluster are child abuse prevention (tls: 1, occurrence: 2), COVID-19 (tls: 8, occurrence: 5), foster care (tls: 9, occurrence: 5), mental health (tls: 9, occurrence: 3), mobile device (tls: 4, occurrence: 2), mobile phone (tls: 7, occurrence: 2), parenting intervention (tls: 5, occurrence: 3), positive parenting skills (tls: 7, occurrence: 2), preterm birth (tls: 4, occurrence: 2), RCT (tls: 4, occurrence: 2), stress (tls: 6, occurrence: 2), and translational research (tls: 7, occurrence: 2). Items in the sixth cluster are behavior problems (tls: 29, occurrence: 11), case series (tls: 2, occurrence: 2), child disruptive behavior (tls: 2, occurrence: 2), community mental health (tls: 9, occurrence: 3), engagement (tls: 14, occurrence: 8), natural helper (tls: 2, occurrence: 2), preschool children (tls: 22, occurrence: 9), single subject design (tls: 6, occurrence: 2), stuttering (tls: 8, occurrence: 5), therapy (tls: 6, occurrence: 4), and treatment outcomes (tls: 13, occurrence: 7). Items in the seventh cluster are adaptations (tls: 5, occurrence: 3), child behavior problems (tls: 17, occurrence: 6), child welfare (tls: 12, occurrence: 7), community intervention (tls: 5, occurrence: 2), cost-effectiveness (tls: 2, occurrence: 2), effectiveness (tls: 10, occurrence: 5), efficacy (tls: 8, occurrence: 3), meta-analysis (tls: 6, occurrence: 5), systematic review (tls: 4, occurrence: 2), and treatment outcome (tls: 22, occurrence: 8). Items in the eighth cluster are conversation analysis (tls: 10, occurrence: 5), emotion coaching (tls: 3, occurrence: 2), emotion regulation (tls: 15, occurrence: 7), emotion socialization (tls: 5, occurrence: 2), externalizing behavior (tls: 3, occurrence: 2), longitudinal (tls: 4, occurrence: 2), reflective functioning (tls: 4, occurrence: 2), socialization (tls: 6, occurrence: 5), television (tls: 4, occurrence: 3), and toddlers (tls: 12, occurrence: 7). Items in the ninth cluster are child development (tls: 2, occurrence: 3), cognitive development (tls: 4, occurrence: 3), developmental delay (tls: 5, occurrence: 2), early intervention (tls: 12, occurrence: 8), language development (tls: 7, occurrence: 4), maternal depression (tls: 7, occurrence: 3), parenting stress (tls: 15, occurrence: 6), and preschoolers (tls: 12, occurrence: 6). Items in the tenth cluster are attrition (tls: 21, occurrence: 7), disruptive behavior (tls: 44, occurrence: 15), dropout (tls: 22, occurrence: 5), follow-up (tls: 12, occurrence: 2), home-based treatment (tls: 1, occurrence: 2), maintenance (tls: 11, occurrence: 2), outcome (tls: 7, occurrence: 2), and treatment (tls: 18, occurrence: 10). Items in the eleventh cluster are child behavior (tls: 10, occurrence: 4), child maltreatment (tls: 22, occurrence: 11), observation (tls: 6, occurrence: 3), preschool depression (tls: 3, occurrence: 2), prevention (tls: 7, occurrence: 2), psychometric properties (tls: 7, occurrence: 2), randomize controlled trial (tls: 3, occurrence: 2), and sensitivity (tls: 3, occurrence: 2). Items in the twelfth cluster are acceptance and commitment therapy (tls: 4, occurrence: 2), anxiety (tls: 7, occurrence: 4), behavior therapy (tls: 4, occurrence: 2), child anxiety (tls: 4, occurrence: 2), oppositional defiant disorder (tls: 11, occurrence: 6), and selective mutism (tls: 6, occurrence: 2). Items in the thirteenth cluster are down syndrome (tls: 1, occurrence: 2), parent–child interaction (tls: 125, occurrence: 96), pragmatic functions (tls: 4, occurrence: 2), repetition (tls: 4, occurrence: 2), sequential analysis (tls: 2, occurrence: 2), temperament (tls: 2, occurrence: 2), and young children (tls: 2, occurrence: 4). Items in the fourteenth cluster are attention deficit/hyperactivity disorder (tls: 4, occurrence: 2), behavior intervention (tls: 2, occurrence: 2), callous-unemotional traits (tls: 3, occurrence: 2), conduct problem (tls: 6, occurrence: 5), families (tls: 3, occurrence: 2), internet-based treatment (tls: 1, occurrence: 2), and trauma (tls: 7, occurrence: 3). Items in the fifteenth cluster are adherence (tls: 11, occurrence: 3), brief treatment (tls: 2, occurrence: 2), competence (tls: 5, occurrence: 2), externalizing behavior problem (tls: 15, occurrence: 6), homework (tls: 7, occurrence: 3), and treatment fidelity (tls: 6, occurrence: 2). Items in the sixteenth cluster are alexithymia (tls: 5, occurrence: 2), attention deficit (tls: 6, occurrence: 2), autism spectrum disorder (tls: 6, occurrence: 2), hyperactivity disorder (tls: 6, occurrence: 2), reliability (tls: 4, occurrence: 2), and validity (tls:4, occurrence:2). The item in the seventeenth cluster is motivation (tls: 2, occurrence: 2) and the item in the eighteenth cluster is behavior observations (tls: 2, occurrence: 2).

When Figure 10B is examined, it is seen that keywords such as treatment, early intervention, oppositional defiant disorder, and joint attention come to the fore in early PCIT research. However, in recent years, language development, developmental delay, barriers, implementation, autism spectrum disorder, mobile phone, home-based treatment, emotional coaching, natural helper, telehealth, obesity and obesity prevention, addiction, parental responsiveness, preschool depression, reflective functioning, emotion regulation, and time-out are the keywords of come to the fore. When Figure 10C is examined, it is seen that the keywords with the highest density are PCIT, dissemination, implementation, treatment, effectiveness, child welfare, behavior problem, autism spectrum disorder, emotion regulation, early intervention, and child maltreatment.

Based on the links listed, it can be interpreted that PCIT has a parent training quality (especially positive parenting skills) and is considered a short-term approach. In addition, adaptation and dissemination studies of PCIT continue around the world, the effectiveness of the studies beyond adaptation is examined, the therapy has an adaptive structure, and therapist acceptance is an important item. It is clear that the effectiveness of PCIT on child behavior and adjustment problems (e.g., externalization problems, anxiety, oppositional defiance, autism spectrum disorder, emotion regulation, child abuse, trauma, down syndrome, child depression, selective mutism, obesity) has been studied. The effect of this intervention on children, as well as the effect of this intervention on parental stress and emotion regulation, has been examined. In addition, it is seen that PCIT has been examined in home-based and web-based formats in recent years (especially with the impact of COVID-19). Thus, it can be interpreted that PCIT has a structure which may be transferable to technology and its application areas have expanded.

## 4. Discussion

According to the bibliometric analysis results, the United States is leading in PCIT studies with a total of 422 publications (Chaffin et al., 2004; Puliafico et al., 2012; Luby, 2013; Carpenter et al., 2014; Kennedy et al., 2016; Rothenberg et al., 2019; Niec et al., 2020; Agazzi et al., 2022). However, with studies conducted in recent years, it is seen that Germany (*n* = 23), New Zealand (*n* = 7), and Australia (*n* = 44) have gained momentum in PCIT studies (Furuzawa et al., 2020; Woodfield et al., 2020; Kamo et al., 2021; Woodfield et al., 2021). Niec et al. (2018) similarly found that PCIT has become widespread in many countries in recent years, including Australia, Germany, Japan, and France. In this context, it can be interpreted that although PCIT is a United States-originated therapy approach, it has been widely and effectively used in many countries of the world. Depending on new trends in PCIT studies from countries, organizations (e.g., Univ New South Wales and Univ Calif Riverside), researchers (e.g., Jane Kohlhoff, Nancy Briggs, Dainelys Garcia, Jason F. Jent, and Hanan Salem), and citation networks (e.g., Larissa N. Niec, Miya L. Barnett, May Yeh) similar change was observed.

Analyses examining keywords found that in recent years language development, developmental delay, implementation, autism spectrum disorder, mobile phone, home-based treatment, emotional coaching, natural helper, telehealth, obesity, obesity prevention, addiction, parental responsiveness, preschool depression, reflective functioning, emotion regulation, time-out, and barriers keywords come to the fore. Barriers to PCIT studies is also an important keyword in the study. According to Dawson-Squibb et al. (2022), South African families may experience difficulties in meeting time, treatment costs, parental mental health, limited psychological resources for parents, and lack of effort in therapy skills. Additionally, research shows infrastructure needed for therapy practice may be costly, and PCIT is primarily applicable for children aged 2–7 years. However, research based on PCIT for toddlers (Girard et al., 2018), and web-based therapy (Comer et al., 2017; Garcia et al., 2021) emerged in the analysis of keywords. As increases in web-based PCIT applications occurred during the pandemic, it can be interpreted that limitations to therapy may be relatively reduced for familes. Barnett et al. (2021) stated that many PCIT therapists switched to internet-based applications during the pandemic process, and 82% of therapists will prefer internet-based applications after the pandemic.

## 5. Limitations

The fact that the keyword used in the research process is searched only in the title is a limitation of the study, as some researchers may publish PCIT research without including PCIT in the title (Chase and Eyberg, 2008; Bagner et al., 2010; Bagner, 2013). Another limitation is that a speech/language intervention called PCIT might have gotten picked up in the search. Using only WoS as a database is a limitation, as some of the scientific literature may not have been access *via* WoS. Specific results of bibliometric analyses vary based on factors such as the search engine used, the keywords, the different ways that author names are listed on the article, and the use of “or” or “and” as a conjunction. Additionally, the search location of the keywords in the study (e.g., the title was used for the present investigation), publication year, language, etc. will impact the availability of bibliometric data. It is considered a limitation of the study that the keywords of (“parent child interaction”) or (“parent–child interaction”) or (“parent child interaction therapy”) or (“parent–child interaction therapy”) or (“pcit”) used during the search of data sources are used in English. This may limit the analysis of publications indexed in the WoS database in other languages. Given the use of VOSviewer for the analyses in this study, another limitation is the method for dealing with repetition of items (i.e., they are depicted in a single cluster and the others are excluded). Errors may be apparent in the names of universities, the included researchers, the topics, and the citations based on the search parameters. Yet, strengths of the current bibliometric analysis include visualizing data such as prominent keywords, countries, universities, researchers, citations, and research topics which reveals general patterns associated with the historical dissemination of PCIT.

## 6. Conclusion

Based on the analysis of bibliometric data of PCIT publications retrieved from the WoS database, it was concluded that PCIT studies have continued to increase since 1970. According to the classifications created, each covering a range of 13 years, the highest rate of publications occurred between 2010 and 2022 (65%). In conclusion, these findings demonstrate that PCIT is a current research topic for the intervention of disruptive behavior problems and other various emotional, behavioral, and physical health concerns observed in children (e.g., emotion regulation, anxiety, selective mutism, trauma, obesity, language and speech problems, developmental delays) from various cultures.

When the studies were classified according to the WoS categories, it was concluded that the five most popular WoS categories in PCIT research were Psychology Developmental, Psychology Clinical, Psychiatry, Family Studies, and Social Work. Similarly, the five most frequent publishers of PCIT studies include Elsevier, Taylor & Francis, Springer Nature, Sage, and Wiley. In terms of WoS publication index categories, it was concluded that the majority of the publications (85%) were in the SSCI index. While most of the published PCIT studies are carried out in the United States, scientific collaborations have recently been established between Australia, New Zealand, Denmark, Netherlands, Taiwan, and Iran. Furthermore, the central organizations where published PCIT studies are conducted include the Univ Florida, Univ Oklahoma, West Virginia University, Griffith Univ, Michigan State Univ, Univ Calif Davis, Univ Massachusetts Dartmouth, Univ Calif Riverside, Florida State Univ, New York Univ, Univ Sydney, Univ New South Wales, and Texas Tech Univ. Developmental Psychology, Topics in Early Childhood Special Education, Journal of Abnormal Child Psychology, Child & Family Behavior Therapy, and Journal of Consulting and Clinical Psychology are the journals in which PCIT studies are published most frequently. Sheila M. Eyberg, Melanie J. Zimmer-Gembeck, Beverly W. Funderburk, Rae Thomas, Larissa N. Niec, Cheryl B. McNeil, Daniel M. Bagner, Miya L. Barnett, Jonathan S. Comer, Amy D. Herschell, Susan G. Timmer, Eva R. Kimonis, Mark Chaffin, Kathleen Armstrong, Anthony J. Urquiza, Nancy M. Zebell, Lisa M. Ware, G. Mahoney, and H. Lyton are prominent researchers based on citations. The most frequently used keywords for PCIT studies are PCIT, dissemination, implementation, treatment, effectiveness, child welfare, behavior problem, autism spectrum disorder, emotion regulation, early intervention, child maltreatment, treatment, early intervention, oppositional defiant disorder, joint attention, language development, developmental delay, barriers, implementation, autism spectrum disorder, mobile phone, home-based treatment, emotional coaching, natural helper, telehealth, obesity, obesity prevention, addiction, parental responsiveness, preschool depression, reflective functioning, emotion regulation, and time-out. While some keywords are related to the structure of PCIT (e.g., evidence-based interventions, home-based intervention, manual-based behavior therapy), some of them are related to application areas (e.g., ADHD, ASD, ODD).

Although PCIT is a subject area in which international scientific collaborations are intense and current, it is also an area in which collaborations continue to be formed around the world. At this point, it can be determined that intercultural adaptations of PCIT, an effective approach for treating emotional and behavioral problem areas experienced by children and their families with both typical development and developmental delay problems, are continuous.

## Author contributions

SU: conceptualization, methodology, software, formal analysis, writing – original draft preparation, and visualization. İS, CM, and EV: validation. İS and SU: investigation. SU, İS, EV, and CM: writing – review and editing. İS and CM: supervision and project administration. All authors contributed to the article and approved the submitted version.

## Conflict of interest

The authors declare that the research was conducted in the absence of any commercial or financial relationships that could be construed as a potential conflict of interest.

## Publisher’s note

All claims expressed in this article are solely those of the authors and do not necessarily represent those of their affiliated organizations, or those of the publisher, the editors and the reviewers. Any product that may be evaluated in this article, or claim that may be made by its manufacturer, is not guaranteed or endorsed by the publisher.

## Footnotes

^1^ https://www.webofscience.com/wos/woscc/summary/23174a73-e08e-4e18-836d-ac8b381ce0e5-5011fa56/relevance/1
